# Ethanol modulates facial stimulation-evoked outward currents in cerebellar Purkinje cells *in vivo* in mice

**DOI:** 10.1038/srep30857

**Published:** 2016-08-04

**Authors:** Mao-Cheng Wu, Yan-Hua Bing, Chun-Ping Chu, De-Lai Qiu

**Affiliations:** 1 Key Laboratory of Cellular Function and Pharmacology of Jilin Province, Yanbian University, Yanji City, Jilin Province, 133002, China; 2 Department of osteology, Affiliated Hospital of Yanbian University, Yanji City, Jilin Province, 133000, China; 3 Department of Physiology and Pathophysiology, College of Medicine, Yanbian University, Yanji City, Jilin Province, 133002, China; 4 Key Laboratory of Natural Resource of the Changbai Mountain and Functional Molecular of the Ministry of Education, Yanbian University, Yanji City, Jilin Province, 133002, China

## Abstract

Acute ethanol overdose can induce dysfunction of cerebellar motor regulation and cerebellar ataxia. In this study, we investigated the effect of ethanol on facial stimulation-evoked inhibitory synaptic responses in cerebellar Purkinje cells (PCs) in urethane-anesthetized mice, using *in vivo* patch-clamp recordings. Under voltage-clamp conditions, ethanol (300 mM) decreased the amplitude, half-width, rise time and decay time of facial stimulation-evoked outward currents in PCs. The ethanol-induced inhibition of facial stimulation-evoked outward currents was dose-dependent, with an IC_50_ of 148.5 mM. Notably, the ethanol-induced inhibition of facial stimulation-evoked outward currents were significantly abrogated by cannabinoid receptor 1 (CB1) antagonists, AM251 and O-2050, as well as by the CB1 agonist WIN55212-2. Moreover, the ethanol-induced inhibition of facial stimulation-evoked outward currents was prevented by cerebellar surface perfusion of the PKA inhibitors H-89 and Rp-cAMP, but not by intracellular administration of the PKA inhibitor PKI. Our present results indicate that ethanol inhibits the facial stimulation-evoked outward currents by activating presynaptic CB1 receptors via the PKA signaling pathway. These findings suggest that ethanol overdose impairs sensory information processing, at least in part, by inhibiting GABA release from molecular layer interneurons onto PCs.

The cerebellar cortex receives a variety of sensory inputs from climbing fibers and mossy fibers, and generates motor-related outputs which are involved in the control of sensory perception, motor coordination, motor learning and fine adjustment of voluntary movement. Acute alcohol overdose impairs cerebellar function and perturbs motor coordination, balance, behavior, speech, and certain cognitive functions^1^,^2^.

The mammalian cerebellar cortex mainly consists of Purkinje cells (PC), molecular layer interneurons (MLIs), granule cells and Golgi cells^3^. The PC plays a critical role in computation in the cerebellar cortex, receiving converging projections from all other cerebellar cortical neurons and providing the sole output from the cerebellar cortex to the deep cerebellar nuclei^4^. There are two types of excitatory afferent inputs; climbing fibers and mossy fibers. Information from climbing fibers activates cerebellar PCs, triggering the firing of complex spikes, while information from mossy fibers travels along parallel fibers and triggers PCs to fire simple spikes^3^,^4^,^5^. MLIs have historically been divided into basket and stellate cells^3^. These cells receive excitatory input from parallel fibers and inhibitory input from other interneurons, and they exert GABAergic inhibition onto PCs^4^,^6^,^7^. Stellate-type MLIs provide dendritic inhibition onto PCs, which may specifically counterbalance parallel fiber excitation in local regions of PC dendrites^8^. In contrast, basket-type MLIs provide powerful and rapid somatic inhibition of PCs, directly impacting PC spiking output by inhibiting the soma and initial segment of these cells^9^,^10^. MLIs are critical for sensory information processing in the cerebellar cortex^11^,^12^,^13^. We previously reported that air-puff stimulation of the ipsilateral whisker pad triggers GABAergic inhibition of PCs, which manifests as strong outward currents in the PC soma and dendrites under voltage-clamp conditions^13^. Notably, the facial sensory stimulation of trigeminal afferents primarily elicits spike firing in MLIs of the cerebellar cortex crus II^12^.

The cerebellum is an important target of the acute action of ethanol. Ethanol-induced alterations of motor coordination, balance, speech and certain cognitive functions are considered to be caused, at least in part, through impairment of cerebellar function^14^. Neonatal ethanol exposure results in dose-dependent impairments in the acquisition and timing of the conditioned eyeblink response, and alters the activity of the cerebellar interpositus nucleus unit and produces a reduction in neuronal numbers, particularly of PCs and granule cells (GCs) in the cerebellar cortex in adult rats^15^. In the cerebellar cortex, PCs are ethanol-sensitive. Acute application of low concentrations of ethanol increases the current-evoked simple spike firing rate, while high concentrations induce a reduction in the simple spike firing rate^16^,^17^. Ethanol has been shown to increase GABAergic transmission onto PCs via enhanced calcium release from presynaptic internal stores and by increasing the intrinsic firing rate of MLIs in rat cerebellar slices^14^. In addition, ethanol increases the frequency of miniature and spontaneous inhibitory postsynaptic currents in PCs and MLIs, and it decreases the amplitude of excitatory postsynaptic potentials in PCs via increased release of GABA^14^,^18^,^19^.

Recently, we found that high concentrations (>20 mM) of ethanol significantly inhibit sensory stimulation-evoked responses. The alcohol produces significant reductions in the amplitude, the area under the curve, the rise time constant and the decay constant of the inhibitory response. Blockade of GABA_A_ receptor activity abolishes these effects of ethanol on the sensory stimulation-evoked inhibitory responses^20^. We previously showed that ethanol affects sensory stimulation-evoked inhibitory responses in the cerebellar cortical molecular layer via the modulation of GABA release from MLIs onto PCs. However, the mechanisms underlying these actions of ethanol in mouse cerebellar PCs are currently unclear.

In the present study, we investigated the effects of ethanol on the facial stimulation-evoked responses in cerebellar PCs in urethane-anesthetized mice, using *in vivo* patch-clamp recordings. We found that ethanol inhibits facial stimulation-evoked inhibitory synaptic currents. Ethanol decreased the amplitude, half-width, rise time and decay time of the currents in a dose-dependent manner. The ethanol-induced inhibition of the facial stimulation-evoked inhibitory synaptic currents was significantly suppressed by CB1 cannabinoid receptor antagonists and an agonist, as well as by a PKA inhibitor. Taken together, our present results suggest that ethanol overdose impairs sensory information processing, at least in part, by inhibiting presynaptic GABA release from MLIs onto PCs. Our findings provide novel insight into the mechanisms of ethanol-induced impairment of cerebellar function.

## Results

### Ethanol inhibits facial stimulation-evoked outward currents in cerebellar PCs

**Figure 1:**
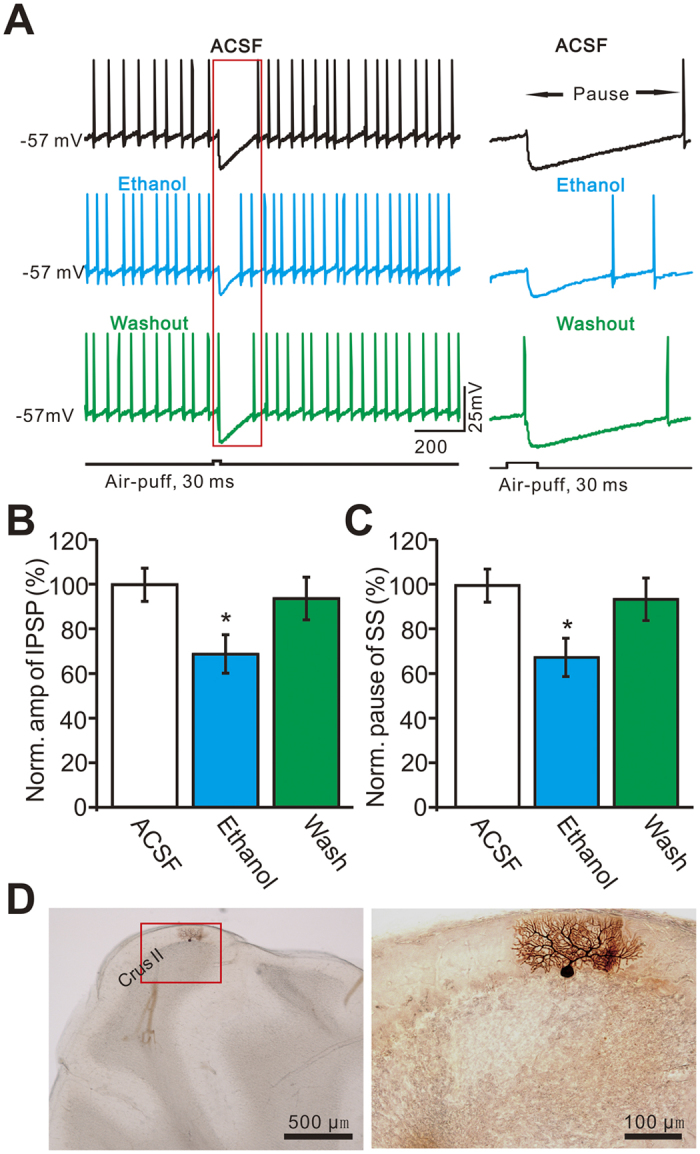
Effect of ethanol on facial stimulation-evoked inhibitory postsynaptic potential (IPSP) of PCs. (**A**) Left, under current-clamp (I = 0), representative traces showing air-puff stimulation (30 ms, 60 psi) evoked IPSPs in a cerebellar PC in ACSF, ethanol (300 mM) and washout. Right, enlarged traces from quadrangle shown in the left panel. (**B**) Summary of normalized amplitude of IPSPs in ACSF, ethanol (300 mM) and washout (n = 18 cells). (**C**) Bar graph shows that the normalized pause of SS firing in ACSF, ethanol (300 mM) and washout (n = 18 cells). (**D**) Left, a photomicrograph depicting the morphology of the PC filled with biocytin. Right panel shows the enlarged microphotograph from quadrangle shown in the left. *P < 0.05 vs ACSF.

Under current-clamp recording conditions (I = 0), 63 PCs (from 63/85 mice) responded to air-puff stimulation of the ipsilateral whisker pad. The mean resting potential of these PCs was 55.2 ± 0.11 mV (n = 63 cells). Consistent with our previous studies^12^,^13^, facial stimulation evoked strong inhibitory postsynaptic potentials (IPSPs), accompanied by a pause in simple spike firing in the somas of PCs under current-clamp conditions (I = 0). The mean amplitude of the IPSPs was 10.4 ± 0.16 mV (n = 63 cells) (Fig. 1A). The mean duration of the pause in simple spike firing was 75.3 ± 3.7 ms (n = 63). Application of ethanol (300 mM) did not significantly change the mean frequency of simple spike firing (Supplementary Figure 1), but it significantly inhibited the facial stimulation-evoked IPSPs; the normalized value of the IPSP amplitude was 68.7 ± 6.2% of baseline (ACSF: 99.9 ± 5.1%, n = 18 cells, *P* = 0.003; Fig. 1B). In addition, the facial stimulation-evoked pause in simple spike firing was decreased in the presence of 300 mM ethanol; the normalized duration of the pause was 67.4 ± 6.4% of baseline (ACSF: 100.0 ± 6.1%, n = 18 cells, *P* = 0.002; Fig. 1C).

**Figure 2:**
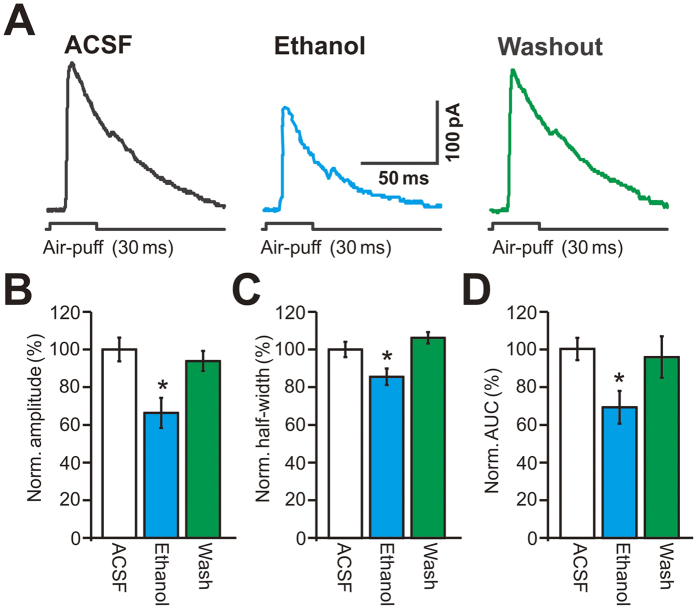
Effect of ethanol on facial stimulation-evoked outward currents in cerebellar PCs. (**A**) Representative traces show the facial stimulation (30 ms, 60 psi) evoked membrane currents in a PC in ACSF, ethanol (300 mM) and washout. (**B–D**) Bar graphs show the normalized amplitude (**B**), half-width (**C**), and AUC (**D**) of outward currents in ACSF, ethanol and washout (n = 18). *P < *0.05 vs* ACSF.

Under voltage-clamp recording conditions (V_hold_ = −70 mV), facial stimulation evoked a sequence of transient inward currents (13.1 ± 2.8 pA, n = 18 cells) followed by strong outward currents (164.1 ± 16.5 pA, n = 18 cells; Fig. 2A). The mean half-width of the outward currents was 51.2 ± 2.1 ms, and the mean area under the curve (AUC) of the IPSCs was 7,848.1 ± 263.9 pA•ms (n = 18 cells; not shown). Application of the GABA_A_ receptor antagonist gabazine (20 μM) blocked the facial stimulation-evoked outward currents, and it also revealed inward currents, indicating that the facial stimulation-evoked outward currents were mediated by the GABA_A_ receptor (Supplementary Figure 1). Perfusion of ethanol (300 mM) reversibly inhibited the facial stimulation-evoked outward currents; the mean normalized amplitude of the outward currents was 66.4 ± 8.0% of baseline (ACSF: 100.0 ± 6.3%, n = 18 cells, *P* = 0.001; Fig. 2B). The mean normalized half-width and the AUC of the outward currents were 85.5 ± 4.4% and 69.1 ± 8.6% of baseline, respectively (half-width: 100.0 ± 4.1%, *P* = 0.028, Fig. 2D; AUC: 100.0 ± 5.9%, *P* = 0.026, Fig. 2E; n = 18 cells). However, in the presence of ethanol (300 mM), the mean amplitude of the inward currents was 98.7 ± 1.2% of baseline (ACSF: 100.0 ± 0.9%, n = 18 cells, *P* = 0.78; data not shown).

**Figure 3:**
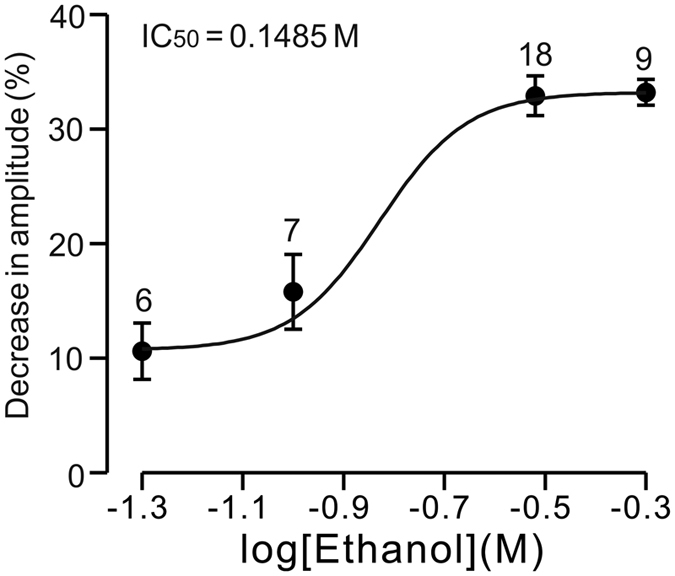
The concentration-response curve shows the ethanol-induced decrease in amplitude of the outward currents in cerebellar PCs. The concentrations of ethanol at 50 mM (n = 6), 100 mM (n = 7), 300 mM (n = 18) and 500 mM (n = 9) were used to examine the effect of ethanol on the facial stimulation-evoked outward currents of PCs. The IC_50_ value obtained from the curve was 148.5 mM. The number of the recorded PCs tested for each concentration indicated near the bars.

The ethanol-induced reductions in the parameters of the facial stimulation-evoked outward currents recovered to baseline after the 20-min washout period. We next examined the dose-dependency of the ethanol-induced inhibition of facial stimulation-evoked outward currents. As shown in Fig. 3, facial stimulation-evoked outward currents were significantly inhibited by 50 mM ethanol (89.4 ± 2.5% of baseline; n = 6 cells), with an IC_50_ of 148.5 mM. The maximum decrease in the amplitude of outward currents produced by 500 mM ethanol was 33.2 ± 1.12% (n = 9 cells). These results indicate that ethanol reversibly inhibits facial stimulation-evoked outward currents in PCs in a dose-dependent manner.

**Figure 4:**
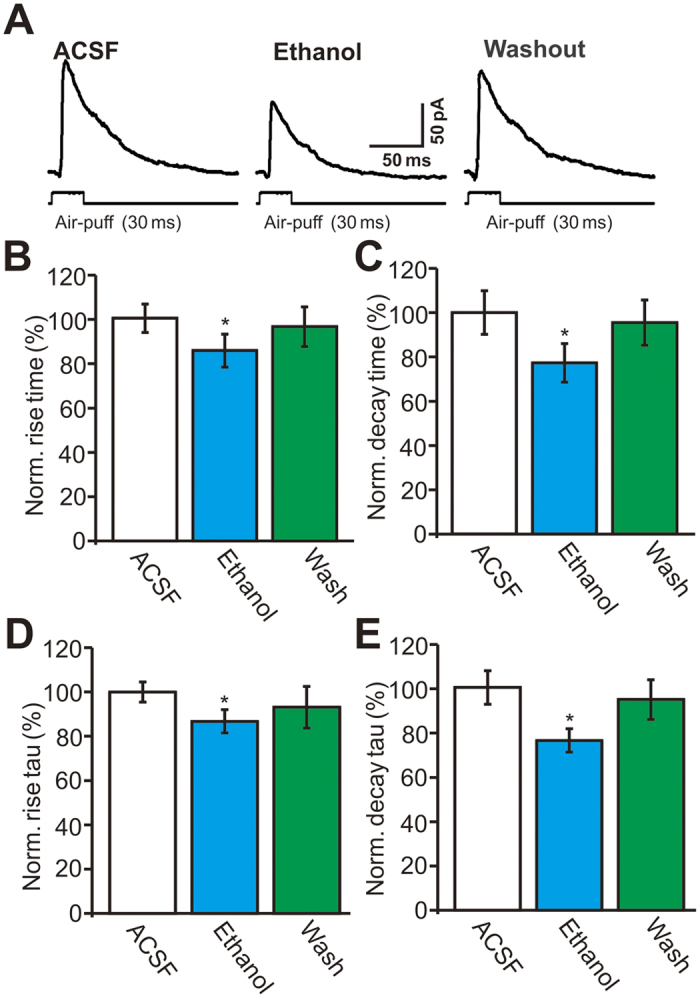
Ethanol modulates dynamics of the facial stimulation-evoked outward currents in cerebellar PCs. (**A**) Representative traces showing facial stimulation (30 ms, 60 psi) evoked outward currents in a PC in ACSF, ethanol (300 mM) and washout. (**B–E**) Bar graphs (n = 18 cells) show the normalized rising time (**B**), decay time (**C**), rise time constant (Tau; (**D**)) and decay time constant (Tau; (**E**)) of the outward currents in treatments of ACSF, ethanol (300 mM) and washout. *P < 0.05 vs ACSF.

We subsequently analyzed the effects of ethanol on the dynamics of facial stimulation-evoked outward currents. Under control conditions, the mean 10–90% rise time was 2.0 ± 0.5 ms (n = 18 cells). Application of 300 mM ethanol induced a significant decrease in the rise time of outward currents to 85.5 ± 7.4% of baseline (ACSF: 100.0 ± 6.4%, *P* = 0.031; Fig. 4A,B). The mean decay time was 109.7 ± 6.9 ms (n = 18 cells) under control conditions, and ethanol (300 mM) decreased the decay time of the outward currents to 77.3 ± 8.7% of baseline (ACSF: 100.0 ± 9.9%, *P* = 0.036; Fig. 4A,C). The rise time constant and decay time constant were 4.2 ± 0.4 ms and 43.7 ± 4.8 ms, respectively, under control conditions. Ethanol (300 mM) decreased the rise time constant to 86.8 ± 5.3% of baseline (ACSF: 100.0 ± 4.6%, *P* = 0.023; Fig. 4A,D), and it decreased the decay time constant to 76.2 ± 5.2% of baseline (ACSF: 100 ± 7.5%, *P* = 0.026; Fig. 4A,E).

### Ethanol-induced inhibition of facial stimulation-evoked outward currents involves activation of the CB1 receptor

**Figure 5:**
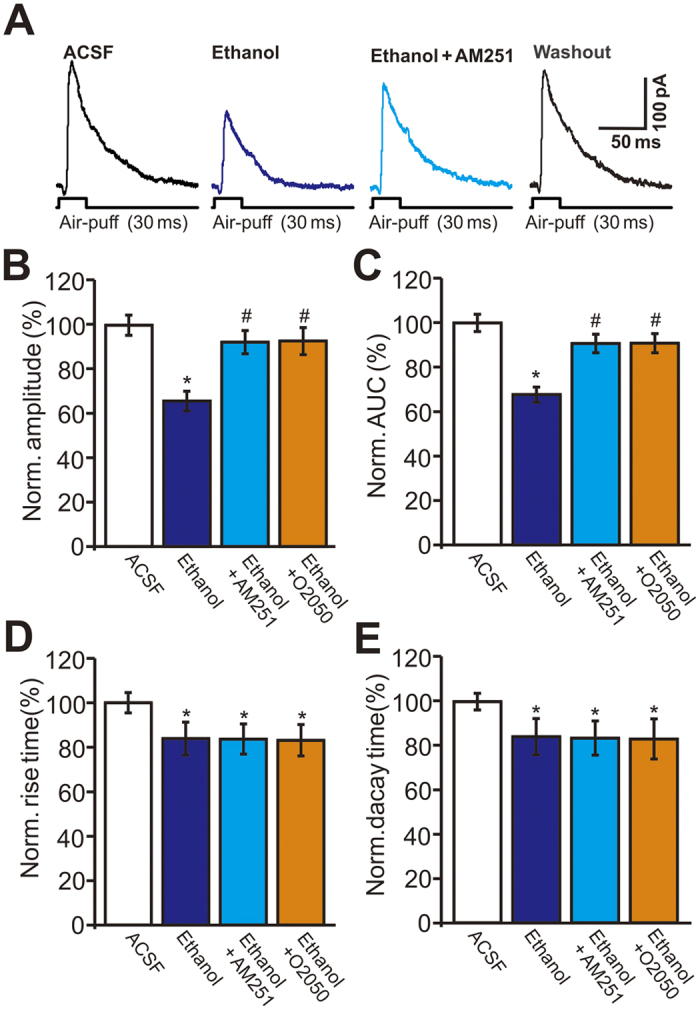
The facial stimulation induced inhibition of the outward currents in PCs was abolished by CB1 receptor antagonists, AM251 and O-2050. (**A**) Representative traces showing facial stimulation (30 ms, 60 psi) evoked outward currents in a PC in treatments of ACSF, ethanol (300 mM), ethanol + AM251 and washout. (**B**) Bar graphs show the normalized amplitude (**B**), AUC (**C**), rise time (**D**) and decay time (**E**) of outward currents in treatments of ethanol, ethanol + AM251, ethanol + O-2050 and washout. n = 6 cells per group. *P < 0.05 vs ACSF; ^#^*P* < 0.05 vs Ethanol.

In the cerebellar cortex, endocannabinoids are known to be generated and released from PCs and MLIs, which are involved in PF–PC and MLI–PC synaptic transmission and plasticity^21^,^22^,^23^. To determine the role of the CB1 receptor in ethanol-induced inhibition of facial stimulation-evoked outward currents in cerebellar PCs, we used the CB1 receptor antagonists AM251 and O-2050. AM251 and O-2050 (not shown) significantly abrogated the ethanol-induced inhibition of the facial stimulation-evoked outward currents (Fig. 5A). In the presence of AM251 and O-2050, ethanol decreased the amplitude of the outward currents to 92.3 ± 5.2% (n = 6) and 92.6 ± 6.1% (n = 6 cells), respectively, of baseline. These changes in the presence of the inhibitors were significantly smaller than those induced by ethanol alone (65.7 ± 4.4% of baseline, n = 12 cells, *P* = 0.002; Fig. 5B). The ethanol-induced decreases in the AUC of the outward currents in the presence of the inhibitors (AM251: 90.8 ± 4.2% of baseline, n = 6 cells; O-2050: 90.9 ± 4.3% of baseline, n = 6 cells) were significantly smaller than the reductions induced by ethanol alone (68.9 ± 4.0% of control, n = 6 cells, *P* = 0.003; Fig. 5C). In addition, blockade of CB1 receptor activity did not prevent the ethanol-induced decrease in rise time or decay time. In the presence of AM251 and O-2050, ethanol decreased the rise time of the outward currents to 83.6 ± 6.8% (n = 6 cells) and 83.1 ± 7.1% (n = 6 cells), respectively, of baseline. These decreases were not significantly different from those induced by ethanol alone (83.8 ± 7.4% of control, n = 12 cells, *P* > 0.05; Fig. 5D). Ethanol decreased the decay time of the outward currents to 83.5 ± 7.7% (n = 6 cells) and 83.1 ± 9.1% (n = 6 cells) of baseline in the presence of AM251 and O-2050, respectively, similar to the changes induced by ethanol alone (84.1 ± 8.2% of control, n = 12 cells, *P* > 0.05; Fig. 5E). These results indicate that activation of CB1 receptors is involved in the ethanol-induced inhibition of the facial stimulation-evoked outward currents.

**Figure 6:**
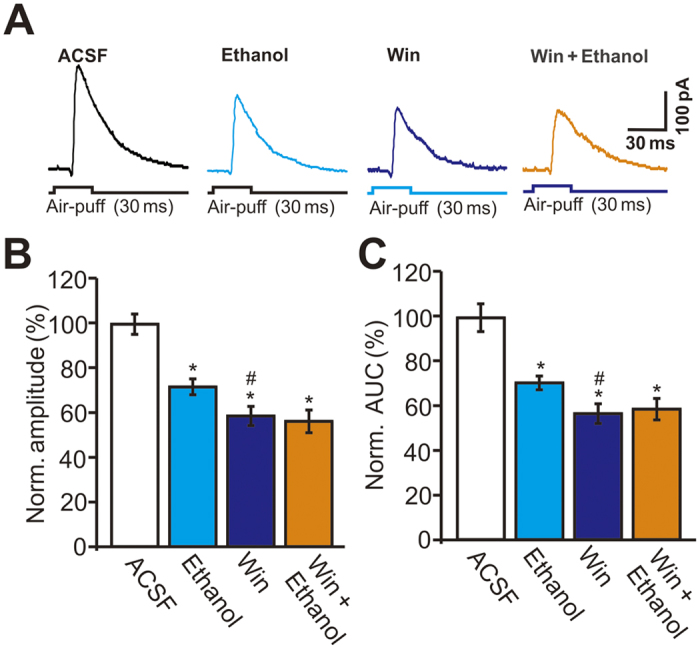
Ethanol failed to inhibit the facial stimulation-evoked outward currents in PCs in the presence of CB1 receptor agonists, WIN55212-2. (**A**) Representative traces showing facial stimulation (30 ms, 60 psi) evoked outward currents in a PC in treatments of ACSF, ethanol (300 mM), WIN55212-2 (5 μM; 10 min), ethanol + WIN55212-2. (**B**,**C**) Summary of data shows the normalized amplitude (**B**) and AUC (**C**) of outward currents in treatments of ACSF, ethanol, WIN55212-2 (5 μM; 10 min), ethanol + WIN55212-2. n = 6 cells per group. *P < 0.05 vs ACSF; ^#^*P* < 0.05 vs Ethanol.

Our findings suggest that pharmacological activation of the CB1 receptor should inhibit facial stimulation-evoked outward currents in PCs. To test this, we examined the effect of the CB1 agonist WIN55212-2 on facial stimulation-evoked outward currents. Perfusion of WIN55212-2 (5 μM) induced significant decreases in the amplitude and AUC of the facial stimulation-evoked outward currents (Fig. 6A). In the presence of WIN55212-2, the normalized amplitude of facial stimulation-evoked outward currents was 58.7 ± 4.3% of baseline, which was significantly smaller than that in ACSF (100.0 ± 4.6%, *P* < 0.001, n = 6 cells) or ethanol alone (71.8 ± 3.6%, *P* = 0.026, n = 6 cells; Fig. 6B), while the normalized AUC was 56.7 ± 4.4% of baseline (100.0 ± 6.2%, n = 6 cells, *P* < 0.001; Fig. 6C), which was also significantly smaller than that in ethanol alone (70.6 ± 3.1%, *P* = 0.036, n = 6 cells; Fig. 6C). Notably, pharmacological activation of the CB1 receptor significantly suppressed the ethanol-induced reductions in the amplitude and AUC of the facial stimulation-evoked outward currents. In the presence of a mixture of WIN55212-2 and ethanol (300 mM), the normalized amplitude of facial stimulation-evoked outward currents was 56.3 ± 5.1% of baseline, which was similar to that in the presence of WIN55212-2 alone (58.7 ± 4.3%, n = 6, *P* > 0.05; Fig. 6B), and the normalized AUC was 58.7 ± 4.8% of baseline, which was not significantly different from that in the presence of WIN55212-2 alone (56.7 ± 4.4%, n = 6 cells, *P* > 0.05; Fig. 6C). These results indicate that pharmacological activation of the CB1 receptor significantly inhibits facial stimulation-evoked outward currents, and that it prevents the ethanol-induced decreases in the amplitude and AUC of the facial stimulation-evoked outward currents.

### PKA activation is required for the ethanol-induced decrease in facial stimulation-evoked outward currents

**Figure 7:**
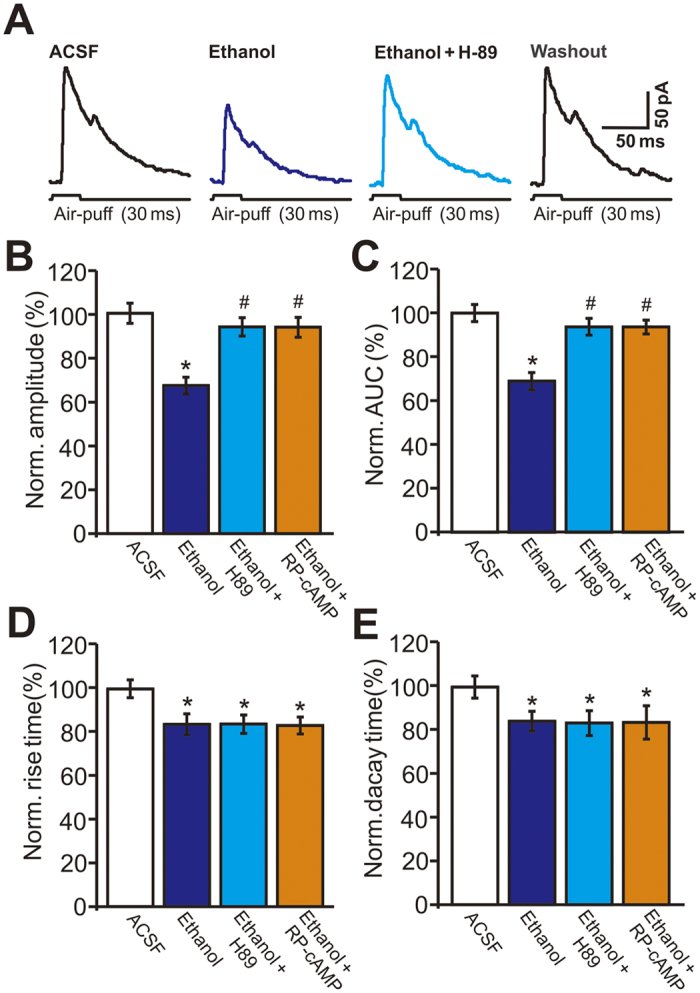
Ethanol inhibits the facial stimulation-evoked outward currents via protein kinase A (PKA) pathway. (**A**) Representative traces showing facial stimulation (30 ms, 60 psi) evoked outward currents in a PC in treatments of ACSF, ethanol (300 mM), ethanol + H-89 (50 μM) and washout. (**B**–**E**) Bar graphs show the normalized amplitude (**B**), AUC (**C**), rise time (**D**) and decay time (**E**) of outward currents in treatments of ethanol, ethanol + H-89, ethanol + RP-cAMP and washout. n = 6 cells per group. *P < 0.05 vs ACSF; ^#^*P* < 0.05 vs Ethanol.

To evaluate the role of PKA in the ethanol-induced inhibition of the facial stimulation-evoked outward currents, we employed two general PKA inhibitors: H-89 (50 μM), which acts at the PKA adenosine triphosphate (ATP)-binding site, and Rp-cAMP (50 μM), which acts at cyclic adenosine monophosphate (cAMP) binding sites^24^. As shown in Fig. 7, both H-89 and Rp-cAMP significantly abrogated the ethanol-induced inhibition of facial stimulation-evoked outward currents. In the presence of H-89 and Rp-cAMP, the ethanol-induced decreases in the amplitude of the outward currents were, respectively, 93.8 ± 4.2% (n = 6) and 93.6 ± 4.5% (n = 6) of control, which were significantly smaller than the decrease induced by ethanol alone (67.2 ± 3.8% of control, n = 12 cells, *P* < 0.001; Fig. 7B). The ethanol-induced decrease in the AUC of the outward currents was also suppressed by H-89 and Rp-cAMP. In the presence of H-89 (50 μM) and Rp-cAMP (50 μM), the ethanol-induced decreases in the AUC of the outward currents were, respectively, 93.7 ± 4.0% and 93.6 ± 3.1% of control, which were significantly smaller than the decrease induced by ethanol alone (68.9 ± 4.1% of control, n = 12 cells, *P* < 0.001; Fig. 7C). However, neither H-89 (50 μM) nor Rp-cAMP (50 μM) suppressed the ethanol-induced decrease in rise time or decay time. In the presence of H-89 and Rp-cAMP, ethanol decreased the rise time of the outward currents to 83.8 ± 4.2% (n = 6) and 83.2 ± 3.9% (n = 6), respectively, of control (rise time: 100.0 ± 4.6%, *P* = 0.026, n = 12), which were not significantly different from the reduction produced by ethanol alone (84.3 ± 4.5% of control, *P* > 0.05; Fig. 7D). Similarly, in the presence of H-89 and Rp-cAMP, ethanol decreased the decay time of the outward currents to 83.4 ± 6.7% (n = 6) and 83.7 ± 7.7% (n = 6), respectively, of control (decay time: 100.0 ± 3.1%, *P* = 0.021, n = 12), similar to the reduction induced by ethanol alone (84.3 ± 4.5% of control, *P* > 0.05, Fig. 7E). Blockade of the PKA pathway significantly suppressed the ethanol-induced decreases in the amplitude and AUC, but failed to block the ethanol-induced decreases in rise time and decay time. These results suggest that ethanol inhibits facial stimulation-evoked MLI–PC GABAergic transmission by affecting the PKA pathway.

**Figure 8:**
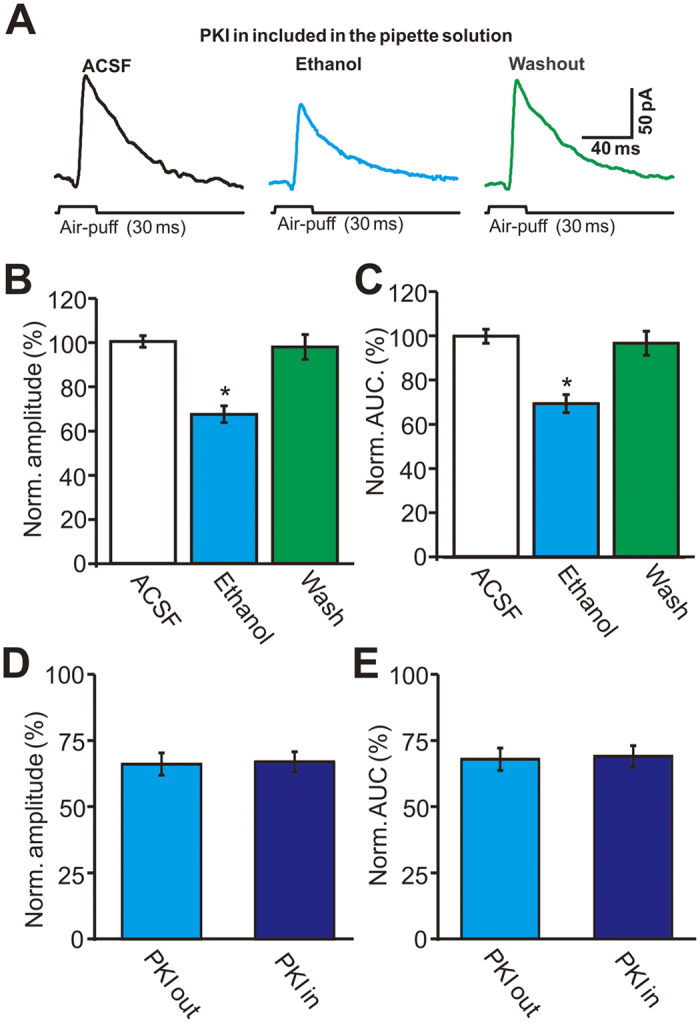
Inhibiting postsynaptic PKA activity did not prevent the ethanol-induced inhibition of the facial stimulation-evoked outward currents. (**A**) With 5 μM PKI in the pipette internal solution, representative membrane currents traces evoked by facial stimulation (30 ms, 60 psi) in a PC in treatments of ACSF, ethanol (300 mM) and washout. (**B**,**C**) Bar graphs show the normalized amplitude (**B**) and AUC (**C**) of the outward currents in treatments of ACSF, ethanol and washout. (**D**,**E**) Bar graphs show that the normalized amplitude (**D**) and AUC (**E**) of the facial stimulation-evoked outward currents using normal pipette internal solution (PKI out) and PKI included pipette internal solution (PKI in). n = 6 cells per group. *P < 0.05 vs ACSF.

To examine whether the PKA antagonists act specifically at the presynaptic terminal and not the postsynaptic neuron, a membrane impermeable PKA inhibitor, PKI (5 μM), was included in the internal pipette solution. In the presence of 5 μM PKI, ethanol (300 mM) significantly decreased the amplitude and AUC of facial stimulation-evoked outward currents (Fig. 8A). The mean normalized amplitude of the outward currents was 67.1 ± 3.8% of baseline (ACSF: 100.0 ± 2.6%, n = 6 cells, *P* < 0.001; Fig. 8B), while the mean normalized AUC of the outward currents was 69.3 ± 4.1% of baseline (ACSF: 99.9 ± 3.2%, *P* < 0.001, n = 6 cells; Fig. 8B,C). With PKI in the internal pipette solution, ethanol decreased the amplitude of the outward currents to 67.1 ± 4.2% of control, similar to the reduction produced by ethanol in normal internal solution (67.8 ± 3.8% of control, *P* > 0.05, n = 6 cells; Fig. 8D). With PKI in the internal pipette solution, ethanol decreased the AUC of the outward currents to 69.3 ± 4.1% of control, similar to the reduction produced by the alcohol in normal internal solution (68.9 ± 4.3% of control, *P* > 0.05, n = 6 cells; Fig. 8E). These findings demonstrate that ethanol decreases facial stimulation-evoked outward currents by reducing GABA release via the activation of presynaptic CB1 receptors, which in turn is dependent on the presynaptic PKA signaling pathway.

## Discussion

The major finding of the present study is that ethanol inhibits facial stimulation-evoked outward currents via the CB1 receptor. Importantly, ethanol-induced inhibition of facial stimulation-evoked outward currents was prevented by extracellular, but not intracellular, administration of a PKA inhibitor. These novel results provide insight into the mechanisms by which ethanol impairs cerebellar function, suggesting that ethanol overdose impairs sensory information processing, at least in part, by inhibiting GABA release from MLIs onto PCs.

### Ethanol inhibits facial stimulation-evoked MLI–PC GABAergic synaptic transmission in cerebellar PCs

Consistent with our previous studies^12^,^13^, air-puff stimulation of the ipsilateral whisker pad evoked a sequence of transient inward currents followed by strong outward currents. The transient inward currents are evoked by parallel fiber excitatory inputs, whereas the outward currents are caused by excitation of MLIs^12^,^13^. The reversal potential of Cl^^−^^ was calculated to be about −53 mV in this study, but the facial stimulation evoked outward currents when PCs were voltage-clamped at −70 mV, which indicates poor clamping of the cerebellar PCs under *in vivo* conditions^12^. Notably, application of the GABA receptor antagonist gabazine abolished IPSCs and revealed EPSCs (Supplementary Figure 1), which confirmed that the evoked outward currents were mediated by the GABA_A_ receptor^12^,^13^. In this study, ethanol inhibited facial stimulation-evoked outward currents, but had a minor effect on the amplitude of the inward currents, indicating that parallel excitatory inputs do not significantly contribute to the ethanol-induced inhibition of outward currents.

It is known that acute or chronic ethanol exposure impairs cerebellar functions, including the control of motor coordination, planning and fine regulation of voluntary movement, motor learning, and cognitive function^1^,^2^. During development and in the adult, ethanol exposure results in dose-dependent impairments in the acquisition and timing of the conditioned eyeblink response, and alters the activity of the cerebellar interpositus nucleus neurons in adult rats^15^. The effects of ethanol on coordination, mood, behavior and sedation have been suggested to be related to its effect on GABAergic function, especially the ability to enhance the inhibitory action of GABA^2^,^25^,^26^,^27^,^28^. Ethanol acutely increases the frequency of miniature and spontaneous IPSCs, and decreases the amplitude of excitatory postsynaptic potentials in PCs in rat cerebellar slices, which suggests that ethanol decreases the excitability of PCs by increasing the amount of GABA released from MLIs onto PCs in the cerebellum^14^. High concentrations of ethanol have been shown to increase the spontaneous release of GABA from MLI axonal terminals onto PCs^14^,^29^,^30^, which can be elicited by increasing the intrinsic firing rate of MLIs through the enhancement of hyperpolarization-activated cationic currents^18^. However, we recently found that high concentrations of ethanol significantly inhibit the GABAergic response, suggesting that ethanol inhibits GABAergic sensory responses in the cerebellar molecular layer^20^.

In this study, we found that ethanol significantly decreased the amplitude of facial stimulation-evoked IPSPs, and shortened the pause in simple spike firing under current-clamp conditions, suggesting that ethanol inhibits facial stimulation-evoked MLI–PC GABAergic synaptic transmission. Ethanol inhibited facial stimulation-evoked outward currents, manifested as decreases in the amplitude, half-width, rise time and decay time of the currents under voltage-clamp conditions, confirming that ethanol decreases facial stimulation-evoked MLI–PC GABAergic synaptic transmission. In addition, the effect of ethanol on the outward currents was influenced by the urethane anesthesia. However, urethane depresses neuronal excitability through activation of barium-sensitive potassium leak conductance, without affecting excitatory glutamate-mediated or inhibitory (GABA_A_ or GABA_B_-mediated) synaptic transmission^31^.

### Ethanol inhibits facial stimulation-evoked outward currents in cerebellar PCs via activation of the endocannabinoid system

Activation of the endocannabinoid system inhibits the release of glutamate from corticostriatal synapses, thereby reducing excitatory postsynaptic currents evoked in medium-sized spiny neurons^32^. It also decreases GABA release from recurrent axon collaterals of medium spiny neurons, thereby reducing inhibitory synaptic transmission intrinsic to the striatum^33^. In the cerebellar cortex, endocannabinoids are released from PCs and MLIs, triggered by activation of mGluR1 receptors following stimulation by spontaneous parallel fiber and/or climbing fiber inputs *in vitro*^34^,^35^ and in living animals^36^. Activation of NMDA receptors on MLIs can induce endocannabinoids release, and may be involved in PF–PC presynaptic LTD in the mouse cerebellar cortex^21^,^22^,^34^,^37^. We recently found that repeated facial stimulation evokes endocannabinoid release from MLIs via activation of NMDA receptors, and that this is involved in MLI–PC GABAergic synaptic LTD, suggesting that endocannabinoids are released from MLIs during sensory stimulation^22^. Consistent with previous studies^22^,^23^, we found that selective agonist of the CB1 receptor significantly inhibits facial stimulation-evoked GABAergic inhibitory responses in PCs, indicating that endocannabinoids function as retrograde messengers in facial stimulation-evoked GABAergic synaptic transmission between MLIs and PCs in the cerebellar cortex. We also demonstrated that the ethanol-induced inhibition of facial stimulation-evoked outward currents was significantly suppressed by CB1 receptor antagonists and an agonist, indicating that ethanol-induced inhibition of facial stimulation-evoked outward currents is mediated, at least in part, by activation of the endocannabinoid system. Our present results suggest that ethanol inhibits facial stimulation-evoked GABA release from MLIs onto PCs via activation of the CB1 receptor. Numerous studies have demonstrated the presence of CB1 receptors on the terminals of GABAergic interneurons and glutamatergic terminals in various brain regions, including the hippocampus^38^,^39^. In cultured hippocampal neurons, ethanol enhances endocannabinoid levels through calcium pathways and depresses miniature postsynaptic current (mEPSC) frequencies via activation of the CB1 receptor, suggesting that endocannabinoids function as retrograde messengers in the ethanol-induced depression of synaptic activities^40^. Moreover, ethanol inhibits the spontaneous activity of pyramidal neurons in the basolateral nucleus of the amygdala through activation of the endocannabinoid system *in vivo* in rats^41^. However, CB1 receptor blockade did not completely abolish ethanol-induced inhibition of facial stimulation-evoked GABAergic response in PCs, suggesting that this effect of ethanol is mediated in part by other mechanisms, such as direct modulation of GABA release or GABA_A_ receptor activity^14^,^29^,^30^.

Several lines of evidence suggest that the physiological effects of acute ethanol exposure are opposed or antagonized by the endocannabinoid system. It is reported that ethanol prevents endocannabinoid-mediated long-lasting disinhibition of striatal output induced by a single stimulation train, and that it reduces LTD induced by low-frequency stimulation of inhibitory synapses. However, high-frequency stimulation, or a higher concentration of CB1 receptor agonist, induces depression of striatal output that is not prevented by ethanol, suggesting that ethanol affects CB1 receptor-mediated signaling in a synapse-specific manner^42^. Moreover, pharmacological activation of CB1 is sufficient to prevent the ethanol-induced pre-synaptic facilitation of GABAergic signaling on pyramidal neurons in the central amygdala^43^. In the cerebellar cortex, ethanol facilitates GABA release from presynaptic terminals onto PCs via a PKA-dependent mechanism that liberates Ca^2+^ from internal stores and does not require endocannabinoid synthesis^29^. However, activation of CB1 blocks the ethanol-induced enhancement of IPSC frequency in cerebellar PCs via the PKA pathway^44^. These results suggest that the endocannabinoid system modulates the effects of ethanol in a region-specific manner. Moreover, it has been reported that acute consumption of ethanol induces an increase in CB1 receptor availability in human subjects^45^. Collectively, our current findings suggest that ethanol enhances facial stimulation-induced endocannabinoid release from MLIs, resulting in an inhibition of facial stimulation-evoked GABAergic transmission at MLI–PC synapses. Our results suggest that changes in endocannabinoid signaling in the cerebellar cortex could be involved in the physiological response to acute alcohol intoxication. In addition, people lose consciousness at an ethanol concentration of 100 mM, while facial stimulation-evoked outward currents are significantly inhibited by 50 mM ethanol. Thus, human drunken behavior could be associated with the suppression of presynaptic GABA release at cerebellar MLI-PC synapses. On the other hand, our results also showed that blockade of CB1 receptor activity did not prevent the ethanol-induced decrease in rise time or decay time, which suggest that ethanol might have some direct effects on GABA receptors kinetics such as reduction of open time in addition to presynaptic effects^26^,^27^.

### Ethanol-mediated reduction of facial stimulation-evoked outward currents in cerebellar PCs is dependent on the PKA signaling pathway

In this study, we examined the role of PKA in ethanol-induced inhibition of facial stimulation-evoked GABAergic responses in cerebellar PCs. Our results show that the ethanol-induced inhibition of facial stimulation-evoked outward currents is prevented by extracellular administration of the PKA inhibitors H-89 and Rp-cAMP, but not by intracellular administration of the PKA inhibitor PKI, suggesting that ethanol inhibits facial stimulation-evoked outward currents by activation of presynaptic CB1 receptors via the PKA signaling pathway. H-89 and Rp-cAMP are functionally distinct PKA inhibitors: H-89 acts at the PKA ATP-binding site, whereas Rp-cAMP binds to the cAMP binding sites, preventing the regulatory subunits from dissociating from the catalytic subunits^24^. Adenylate cyclase and PKA are downstream targets of G_i/s_ proteins, which play an essential role in ethanol-enhanced spontaneous GABA release in cerebellar PCs^43^. It has been reported that the PKA antagonist Rp-cAMP inhibits baseline spontaneous GABA release, which is consistent with the established role for PKA in neurotransmitter release^43^. In addition, similar effects of a PKA antagonist on baseline spontaneous GABA release are seen in the hippocampus and hypothalamus^46^,^47^. Our present results show that both H-89 and Rp-cAMP significantly prevent ethanol-induced inhibition of facial stimulation-evoked outward currents in PCs, indicating that adenylate cyclase and PKA play important roles in ethanol-induced inhibition of facial stimulation-evoked outward currents in PCs. This suggests that ethanol inhibits the facial stimulation-evoked outward currents by activation of presynaptic CB1 receptors via the PKA signaling pathway. The CB1 receptor is coupled to Gi and inhibits adenylate cyclase activity, reducing the production of cAMP and thereby attenuating the activity of PKA^48^, which causes the inhibition of presynaptic voltage-sensitive calcium channels, and the subsequent reduction of evoked synaptic transmission^49^. Therefore, PKA acts at the neurotransmitter release machinery to regulate synaptic transmission^50^.

There is considerable evidence connecting the adenylate cyclase/PKA pathway to the effects of ethanol^51^,^52^. Through a PKA-dependent mechanism, *in vivo* exposure to ethanol induces a long-lasting potentiation of GABAergic synapses in the ventral tegmental area^53^. Some adenylate cyclase isoforms have been linked to ethanol by biochemical, electrophysiological, and behavioral studies in transgenic mice^54^. At the behavioral level, a reduction in PKA signaling affects alcohol consumption and the sensitivity to the sedative effects of alcohol^55^,^56^,^57^,^58^. Therefore, there is evidence of the adenylate cyclase/PKA pathway playing an important role in multiple ethanol actions, extending from the molecular to the behavioral level. Because a membrane impermeable PKA antagonist in the internal pipette solution did not prevent ethanol-induced inhibition of facial stimulation-evoked outward currents, we are confident that this PKA effect is presynaptic. Although this work provides a link between ethanol-enhanced GABA release and the adenylate cyclase–PKA pathway, more research is needed to determine how ethanol interacts with this pathway.

## Methods

### Anesthesia and surgical procedures

The anesthesia and surgical procedures have been described previously^11^,^13^. In brief, the experimental procedures were approved by the Animal Care and Use Committee of Jilin University and were in accordance with the animal welfare guidelines of the U.S. National Institutes of Health. The permit number is SYXK (Ji) 2007-0011. Eighty five adult (6–8-week-old) HA/ICR mice were anesthetized with urethane (1.3 g/kg body weight i.p.). On a custom-made stereotaxic frame, a watertight chamber was created and a 1–1.5 mm aperture was drilled on the skull to expose the cerebellar surface corresponding to Crus II. The brain surface was constantly superfused with oxygenated artificial cerebrospinal fluid (ACSF: 125 mM NaCl, 3 mM KCl, 1 mM MgSO_4_, 2 mM CaCl_2_, 1 mM NaH_2_PO_4_, 25 mM NaHCO_3_, and 10 mM D-glucose) with a peristaltic pump (Gilson Minipulse 3; Villiers, Le Bel, France) at 0.4 ml/min. Rectal temperature was monitored and maintained at 37.0 ± 0.2 °C using body temperature equipment.

### Electrophysiological recording and facial stimulation

*In vivo* whole-cell recordings from PCs were performed with an Axopatch-200B amplifier (Molecular Devices, Foster City, CA) in 63/85 mice. Twenty-two mice were failed to obtain whole-cell recordings from PCs, and each of 63 mice was recorded one PC for further experiment. The signals of PC whole-cell recordings were acquired through a Digidata 1440 series analog-to-digital interface on a personal computer using Clampex 10.3 software. Patch pipettes were made with a puller (PB-10; Narishige, Tokyo, Japan) from thick-wall borosilicate glass (GD-1.5; Narishige). Patch electrodes (4–6 MΩ) contained a solution of the following composition (in mM): potassium gluconate 120, HEPES 10, EGTA 1, KCl 5, MgCl_2_ 3.5, NaCl 4, biocytin 8, Na_2_ATP 4, and Na_2_GTP 0.2 (pH 7.3 with KOH, osmolarity adjusted to 300 mOsm). For blocking postsynaptic PKA in some experiments, protein kinase inhibitor-(6–22) amide (PKI) was included in pipette internal solution. The whole-cell recordings from PCs were performed at depths 150–200 μm under pia mater membrane, and identified by regular spontaneous SSs accompanied with irregular CSs, and confirmed by biocytin histochemistry (Fig. 1D)^13^. In addition, GABA_A_ receptor antagonist, gabazine (SR95531, 20 μM) was applied in some experiment for examining the properties of the outward currents (Supplementary Figure 1).

Facial stimulation was performed by air-puff (30 ms, 60 psi) of ipsilateral whisker pad through a 12-gauge stainless steel tube connected with a pressurized injection system (Picospritzer^®^ III; Parker Hannifin Co., Pine Brook, NJ). The air-puff stimulations of was controlled by a personal computer, which were synchrinized with the electrophysiologial recordings and delivered at 0.05 Hz via a Master 8 controller (A.M.P.I., Jerusalem, Israel) and Clampex 10.3 software (Molecular Device, Foster City, CA).

After the experiments, the whole brain was removed and fixed in 4% paraformaldehyde in 0.1 PBS (pH 7.4) at 4 °C for 24 hours. Slices were cut in the sagittal plane at 200 μm using a vibratome (NVSLM1, Campden Instruments LTD, Loughborough, England), and washed with PBS. The tissue was reacted overnight with an avidin-biotin complex (ABC Elite kit; Vector Laboratories, Burlingame, CA) at 4 °C. Finally, biocytin binding was visualized by 3,3′-diaminobenzidine tetrahydrochloride histochemistry.

### Chemicals

The reagents included and Rp-adenosine 3′,5′-cyclic monophosphorothioate triethylammonium salt hydrate (Rp-cAMP); N-(piperidin-1-yl)-5-(4-iodophenyl)-1-(2,4-di-chlorophenyl)-4-methyl- 1H-pyrazole-3-carboxamide (AM251) and 6aR, 10aR-3-(1-Methanesulfonylamino-4-hexyn-6-yl)-6a,7,10,10 atetrahydro-6,6,9-trimethyl-6H- dibenzo[b,d]pyran (O-2050), blockade of endocannabinoid CB1 receptors; (R)-(+)-[2,3-dihydro-5-methyl-3-(4-morpholinylmethyl) pyrrolo[1,2,3-de]-1,4-benzoxazin-6-yl] -1-naphthalenylmethanone mesylate (WIN55212-2), CB1 receptor agonist; protein kinase inhibitor-(6-22) amide (PKI) and H89, protein kinase inhibitor; NBQX, (2,3-dioxo-6-nitro-1,2,3,4-tetrahydrobenzo[f] quinoxaline-7- sulfonamide) and gabazine (SR95531) hydrobromide (6-imino-3-(4-methoxyphenyl)-1 (6H)-pyridazinebutanoic acid hydrobromide). All chemicals were purchased from Sigma-Aldrich (Shanghai, China). The drugs were dissolved in ACSF, and applied directly onto the cerebellar surface by a peristaltic pump (0.5 ml/min).

### Data analysis

The electrophysiological data were analyzed using Clampfit 10.3 software. Values are expressed as the mean ± S.E.M. Student’s paired *t*-test and one-way ANOVA (SPSS software; Chicago, IL) were used to determine the level of statistical significance between groups of data. *P*-values below 0.05 were considered to indicate a statistically significant difference between experimental groups.

## Additional Information

**How to cite this article**: Wu, M.-C. *et al*. Ethanol modulates facial stimulation-evoked outward currents in cerebellar Purkinje cells *in vivo* in mice. *Sci. Rep.* **6**, 30857; doi: 10.1038/srep30857 (2016).

## Supplementary Material

Supplementary Information
