# Supplementary material for: Ethanol modulates facial stimulation-evoked outward currents in cerebellar Purkinje cells in vivo in mice
Source: Sci Rep. 2016 Aug 4;6:30857. doi: 10.1038/srep30857 (PMC4973232; doi:10.1038/srep30857)
Supplement: Supplementary Information [file srep30857-s1.pdf]

Supplementary information

Title： Ethanol modulates facial stimulation-evoked outward currents in cerebellar Purkinje cells in vivo in mice

Mao-Cheng Wu[1,3,5], Yan-Hua Bing [1,2,5], Chun-Ping Chu[1*] and De-Lai Qiu[1,2,4*]

[1] Key Laboratory of Cellular Function and Pharmacology of Jilin Province, Yanbian University, Yanji City, Jilin Province, China

[2] Department of Physiology and Pathophysiology, College of Medicine, Yanbian University, Yanji City, Jilin Province, 133002, China

[3] Department of osteology, Affiliated Hospital of Yanbian University, Yanji City, Jilin Province, 133000, China

[4] Key Laboratory of Natural Resource of the Changbai Mountain and Functional Molecular of the Ministry of Education, Yanbian University, Yanji City, Jilin Province, 133002, China

5These authors contributed equally to this work.

Running title: Ethanol modulates sensory response in vivo in mice

Author for correspondence:

De-Lai Qiu, M.D., Ph.D

Cellular Function Research Center, Yanbian University, Yanji City, Jilin Province, China, 133002

Department of Physiology and Pathophysiology, College of Medicine, YanBian University 977, GongYuan Road, Yanji City, Jilin Province, China,133002

Tel: +86-(0)433-2435051; Fax: +86-433-2435051; E-mail: dlqiu@ybu.edu.cn

Chun-Ping Chu, M.D., Ph.D

Cellular Function Research Center, YanBian University, 977, GongYuan Road, Yanji City, 133002, Jilin Province, China

Tel: +86-433-2435050; Fax: +86-433-2435051; E-mail: cpchu@ybu.edu.cn

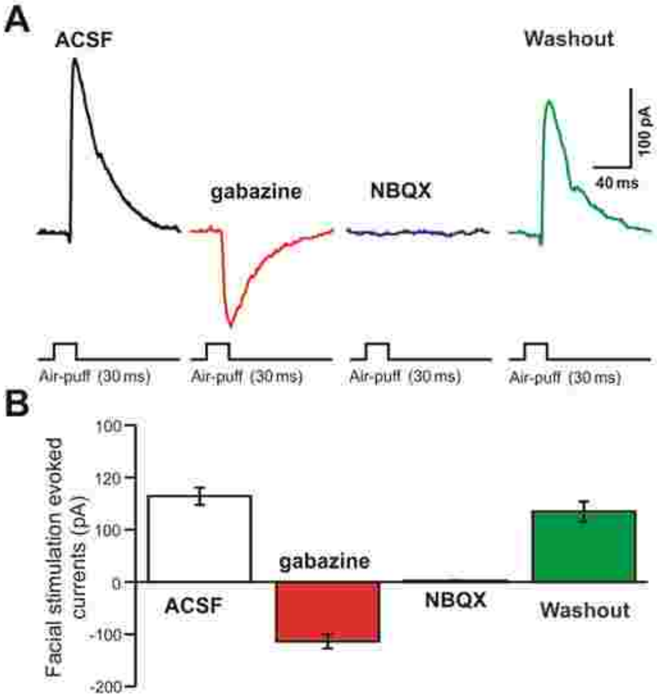

Supplementary Figure 1. Pharmacological properties of the facial stimulation evoked outward currents of cerebellar PCs. (A) Representative traces showing the facial stimulation-evoked outward currents of a PC in treatments of ACSF, gabazine (SR95531, 20 μM), NBQX (50 μM) and washout. (B) Bar graph (n = 5) shows the mean facial stimulation-evoked outward currents of a PC in treatments of ACSF, gabazine, NBQX and washout.

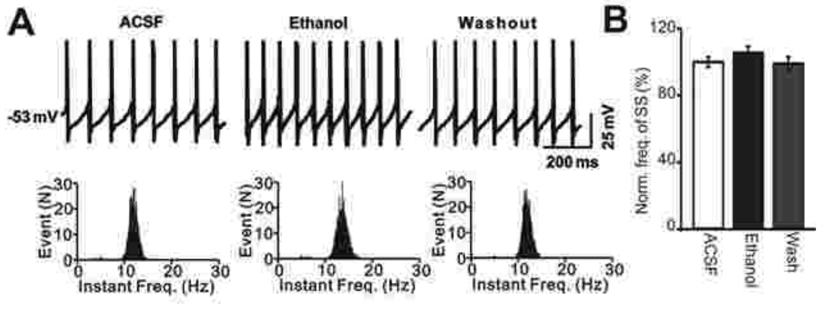

Supplementary Figure 2. Ethanol affected simple spike activity of cerebellar PCs in vivo. (A) Upper, Representative traces showing the spontaneous simple spike activity of a PC in treatments of ACSF, ethanol (300 mM) and washout. Lower, histograms show the instant frequency of simple spikes in treatments of ACSF, ethanol (300 mM) and washout. (B) Bar graph (n = 12) shows the effect of ethanol on the frequency of simple spike activity.
